# Paradigm shift in Parkinson's disease: using continuous telemonitoring to improve symptoms control. Results from a 2-years journey

**DOI:** 10.3389/fneur.2024.1415970

**Published:** 2024-06-06

**Authors:** Spyridon Konitsiotis, Athanasia Alexoudi, Panagiotis Zikos, Christos Sidiropoulos, George Tagaris, Georgia Xiromerisiou, Konstantinos Tsamis, Nicholas Kostikis, Foivos Kanellos, Adamantios Ntanis, Spyridon Kontaxis, George Rigas

**Affiliations:** ^1^University Hospital of Ioannina and Faculty of Medicine, School of Health Sciences, University of Ioannina, Ioannina, Greece; ^2^Department of Neurology, Neurological Institute of Athens, Athens, Greece; ^3^Department of Neurology, 251 Hellenic Air Force Hospital, Athens, Greece; ^4^Department of Neurology, Michigan State University, East Lansing, MI, United States; ^5^Department of Neurology, General Hospital of Athens, “Georgios Gennimatas”, Athens, Greece; ^6^Department of Neurology, University Hospital of Larissa, Larissa, Greece; ^7^Department of Physiology, Faculty of Medicine, School of Health Sciences, University of Ioannina, Ioannina, Greece; ^8^PD Neurotechnology Ltd., Ioannina, Greece

**Keywords:** telemonitoring, real-world data, objective motor assessment, longitudinal analysis, patient satisfaction

## Abstract

**Introduction:**

Conventional care in Parkinson's disease (PD) faces limitations due to the significant time and location commitments needed for regular assessments, lacking quantitative measurements. Telemonitoring offers clinicians an opportunity to evaluate patient symptomatology throughout the day during activities of daily living.

**Methods:**

The progression of PD symptoms over a two-year period was investigated in patients undergoing traditional evaluation, supplemented by insights from ambulatory measurements. Physicians integrated a telemonitoring device, the PDMonitor^®^, into daily practice, using it for informed medication adjustments.

**Results:**

Statistical analyses examining intra-subject changes for 17 subjects revealed a significant relative decrease of −43.9% in the device-reported percentage of time spent in “OFF” state (from 36.2 to 20.3%). Following the 24-month period, the majority of the subjects improved or exhibited stable symptom manifestation. In addition to positively impacting motor symptom control, telemonitoring was found to enhance patient satisfaction about their condition, medication effectiveness, and communication with physicians.

**Discussion:**

Considering that motor function is significantly worsened over time in patients with PD, these findings suggest a positive impact of objective telemonitoring on symptoms control. Patient satisfaction regarding disease management through telemonitoring can potentially improve adherence to treatment plans. In conclusion, remote continuous monitoring paves the way for a paradigm shift in PD, focusing on actively managing and potentially improve symptoms control.

## 1 Introduction

Parkinson's disease (PD) is the second most prevalent neurodegenerative disorder, characterized by a progressive deficiency of dopamine in the brain (1). While the clinical phenotype of PD encompasses various non-motor symptoms like cognitive decline, impulsive behavior, depression, and autonomic nervous system disorders, the primary focus in diagnosing PD revolves around the presence of core motor symptoms, including bradykinesia, muscle stiffness, and tremor (2). Currently, the process of diagnosing and making treatment decisions is conducted through clinical examinations, scales, and patient-reported outcomes (3). To date, therapeutic interventions primarily rely on dopamine replacement drugs, with levodopa being particularly effective in the initial phases of the disease (4).

Nevertheless, disease progression differs from patient to patient and advances at different rates. Conventional care faces limitations due to the significant time and location commitments needed for regular assessments, and it depends on the expertise of physicians, lacking quantitative measurements (5). The accuracy of disease assessment may also be impeded by recall bias and difficulties in patients effectively conveying their symptoms (6). Moreover, the management of PD presents a complex interplay between symptom control and disease progression. Currently, there are no therapies available that can affect the progression of PD. The primary emphasis of medical care is to control the motor symptoms through the use of drugs (7). However, prolonged use of medication leads to significant motor complications, such as limited mobility during the “OFF” period, wearing-off and end-of-dose phenomena, necessitating further treatment modifications (8). To address this unmet need, the utilization of wearable devices, which have become instrumental in telemonitoring, has facilitated the continuous objective measurement of PD symptoms (9).

Telemonitoring offers clinicians an opportunity to evaluate patient symptomatology throughout the day during activities of daily living, to assess response to therapy during long periods of months and years, and to improve follow-up care (10). The concept of continuous telemonitoring for symptoms aligns with the existing standard of care and is not a novel concept in the context of PD (11). Such transformative approaches for PD management pave the way for a paradigm shift aimed at actively managing and potentially improving symptoms control. In this study, the progression of PD symptoms over a two-year observation period in patients undergoing traditional evaluation, combined with insights derived from objective ambulatory measurements is investigated. To the authors' knowledge, this marks the first study involving longitudinal objective real-world data collected at non-clinical settings.

## 2 Materials and methods

### 2.1 Telemonitoring system

The PDMonitor^®^ ecosystem, manufactured by PD Neurotechnology Ltd., is a class IIa medical device for continuous home monitoring of Parkinson's disease patients. It comprises a base (or SmartBox), monitoring devices, mounting accessories, a mobile app, a physician online dashboard, and a cloud service provide an environment for long-term remote PD monitoring. The system includes five wearable sensing devices with motion sensors and accessories for attachment to particular body regions, as well as a SmartBox for collecting and uploading data. For a more in-depth exploration of the ecosystem, the interested reader is directed to (12).

To measure everyday activities and device reported outcomes (DROs) associated with PD, the system uses digital signal processing and machine learning to assess raw movement signals. The system automatically detects waist and limb device positioning throughout waking hours. System output includes heatmaps of symptom severity for a 30-min interval and plots of average symptom intensity for any time of day. The DROs include the percentage of time in “OFF” state (OFF), the percentage of time with dyskinesia (DYS), and the percentage of time in “ON” state (ON), that is defined as 100-OFF-DYS. Moreover, the system provides DROs associated with the unified Parkinson's disease rating scale (UPDRS). As it was suggested by NICE in 2023 (13), the system presents a novel way to remote PD monitoring, giving useful information associated with the antiparkinsonian therapy.

### 2.2 Dataset

A cohort of 20 patients who utilized the telemonitoring device in Greece for 2 years formed the basis of this study. These individuals worn the wearable sensors over multiple days, allowing averaged symptom data extraction. To guarantee the inclusion of high-quality data, DROs corresponding to single-day recordings were excluded, leading to the final cohort comprising 17 subjects. The demographic data of the participants are provided in Table 1. Consistent with applicable privacy laws across the world, no identifiable protected health information (PHI) was extracted, accessed, or used during the course of the study. Pursuant to the USA Health Insurance Portability and Accountability Act (HIPAA) of 1996 with updated provisions (14), the EU General Data Protection Regulation (GDPDR) of 2018 (15), our study used de-identified or anonymous data and therefore does not require institutional review board (IRB) approval or waiver of authorization. Physicians incorporated DROs into their daily practices, relying on this tool to make informed decisions about medication adjustments. Notably, patients with advanced therapies such as Deep Brain Stimulation (DBS) and infusion pumps were excluded from this specific analysis on medication management.

**Table 1:** Demographic data of the participants^a^.

Sex	10 females, seven males
Age	64.3 (10.4) years
Years with disease	8.9 (6.6) years
LEDD (baseline)	799.6 (451.2) mg
LEDD (2-years)	1,055.2 (453.8) mg

LEDD, L-dopa equivalent daily dose. ^a^Total values are presented as “mean (standard deviation)”.

### 2.3 Statistical analysis

In this study evaluating the progression of motor core symptoms in PD, a paired *t*-test was employed to assess changes between baseline (0 months) and the end of the study (24 months) for each participant individually. This analysis focused on within-subject differences, providing insights into the efficacy of the intervention over the study period. Additionally, a linear mixed-effects model was employed to analyze the longitudinal data, incorporating random slopes and random intercepts to account for variations among subjects. The model allowed for the examination of individual trajectories over time while considering both fixed effects, such as time points, and random effects, capturing subject-specific deviations from the overall trend. This comprehensive statistical approach facilitated the exploration of both within-subject changes and inter-subject variability, providing a robust analysis of the impact of the intervention on motor core PD symptoms over the 24-month study duration. In this study, the significance threshold was set to *p* < 0.05.

Questionnaires, employing Likert scales and qualitative inquiries, were also administered to provide valuable insights into the multifaceted clinical benefits of the telemonitoring approach, encompassing patient satisfaction, medication efficacy, patient-physician interactions, and the overall perceived advantages of remote monitoring in the management of PD. Questionnaires were administrated before, during, and after the use of telemonitoring system, allowing for multiple responses from each subject.

## 3 Results

**Figure 1:**
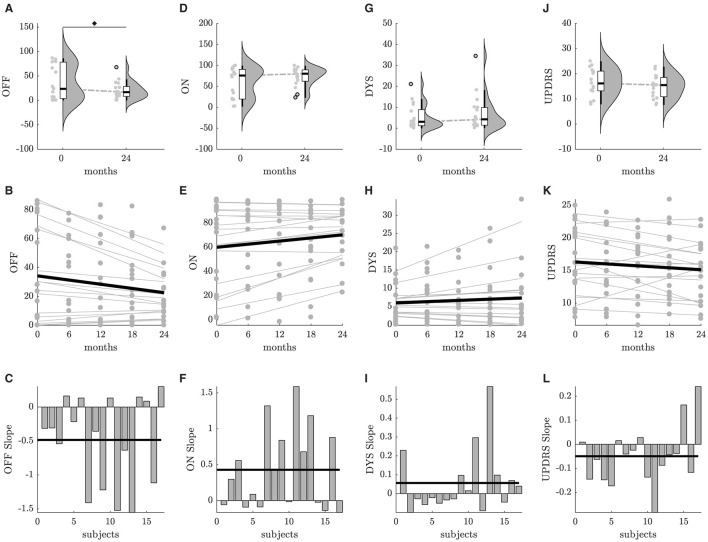
Findings from statistical analyses concerning different DROs. **(A–C)** OFF, **(D–F)** ON, **(G–I)** DYS, **(J–L)** UPDRS. The first row displays a combination of boxplots, scatter plots and violin plots for data collected at both 0 and 24 months. Statistically significant differences between groups are marked with an asterisk. The second row shows plots with individually fitted lines for longitudinal data. The average of these individual thick lines is represented by a heavy black line. The third row illustrates barplots with the slope values across all subjects. The mean slope is indicated with a heavy black line.

Figure 1 illustrates the findings of the statistical analyses for various DROs. As depicted in Figure 1A, a statistically significant decrease in the percentage of OFF (−15.9, or −43.9%) was observed at the end of the study compared to baseline. The intra-subject differences between 0 months and 24 months indicate that telemonitoring contributes significantly to OFF improvement. While there is also a discernible ascending trend of ON outcome (Figure 1D), the statistical significance was not attained. This can be attributed both to the small sample size and the increase of dyskinesia in some patients (Figure 1G). No statistical differences were found for device-reported UPDRS, highlighting the stability of motor core symptoms (Figure 1J).

Results from linear mixed-effects models depict the influence of telemonitoring on the motor core PD symptoms throughout the 24-month study period. Among various DROs, the temporal factor exhibited a statistically significant effect on OFF (mean slope = −0.48, *p* < 0.05), as illustrated in Figure 1B. Although the effect of time was not significant concerning other DROs, subject-specific slopes suggest a notable increase in ON (Figure 1F), while UPDRS either decreased or remained relatively constant across the majority of the subjects (Figure 1L). Only a limited number of participants exhibited exacerbation in terms of dyskinesia (Figure 1I).

The findings underscore the utility of telemonitoring in comprehensively evaluating and understanding the dynamic changes in PD symptoms over an extended observational timeframe. Following a 24-month period utilizing the telemonitoring system, the majority of the subjects improved or exhibited consistent symptom manifestation, and a reduction in the number of patients lacking proper control of their motor symptoms was achieved. The considerable significance of time as a determinant in the progression of PD symptoms when employing the telemonitoring system in patients with PD undergoing traditional evaluation, proves the efficacy of the intervention.

**Figure 2:**
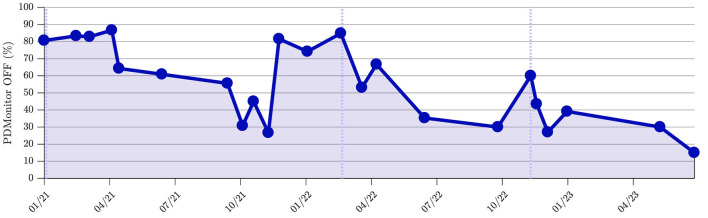
An example of device-reported OFF evolution for a patient. Vertical dashed lines indicate adjustments of treatment regimen.

Figure 2 depicts an example of OFF evolution for a patient. The patient, a 71-year-old female diagnosed with PD a decade ago and treated with levodopa and rotigotine patches for the last 5 years, had OFF phenomena lasting more than 3 h in the last months of 2020. At the beginning of 2021, the patient was recommended to use the telemonitoring system to evaluate symptom fluctuation during the day and the intensity and duration of wearing OFF phenomena. Adjustments in dosing intervals of levodopa (daily dose 475 mg) and dietary habits resulted in a reduction in reported OFF periods and an enhancement in ON periods after three months. These improvements were sustained for more than 6 months without increasing the daily dose. However, escalation in daytime OFF periods led to a decision by the physician to change the treatment regimen by increasing the daily dose from 475 mg of levodopa to 750 mg. The improvement was sustained for 9 months, after which deterioration in gait occurred again. As this was corroborated by the physician during the subsequent visit, further adjustments to the treatment regimen were implemented. Firstly, the discontinuation of the rotigotine patch was decided due to the observed onset of impulse control disorder. Additionally, guided by the insights provided by the DROs, the dosage of levodopa was increased from 750 to 1,250 mg. The efficacy of the treatment was reassessed in the subsequent months using the telemonitoring device, confirming that the patient's condition was adequately managed.

Table 2 presents the results from questionnaires regarding the satisfaction levels of patients. There is a notable increase in the percentage of satisfied patients, rising from 26.9 to 38.5%, regarding the effectiveness of the medication both before and after the utilization of telemonitoring, respectively. Furthermore, the proportion of very satisfied patients experienced a substantial surge, escalating from 7.7 to 23.1%. In contrast, the dissatisfied group exhibited a decline, decreasing from 46.2 to 34.6%. Moreover, upon analyzing the responses from patients concerning the perceived advantages of remote monitoring, a significant majority, constituting 81.5%, acknowledged the beneficial impact of telemonitoring (Table 3). Additionally, 44.4% of respondents reported an improvement in their condition attributable to telemonitoring, while 37.0% indicated that their condition remained unchanged (Table 4). Notably, a substantial proportion of participants (81.5%) conveyed that telemonitoring enhanced communication with their physicians (Table 5). Improved patient-physician interactions and enhanced perceived effectiveness of the medication underscore the positive impact of telemonitoring in the context of PD management.

**Table 2:** **Q1**:“How satisfied are you with the effectiveness of your medication prior to using the PDMonitor^®^?” **Q2**:“How satisfied are you with the effectiveness of your medication after using the PDMonitor^®^?”

**Choices**	**Q1 (%)**	**Q2 (%)**
Very satisfied	7.7	23.1
Satisfied	26.9	38.5
Neither	46.2	34.6
Dissatisfied	15.4	3.8
Very dissatisfied	3.8	0
Didn't answer	0	0

**Table 3:** “To what degree do you agree that the use of PDMonitor^®^ has helped you up to this point?”

**Choices**	**Answers (%)**
Strongly agree	59.3
Agree	22.2
Undecided	7.4
Disagree	11.1
Strongly disagree	0
Didn't answer	0

**Table 4:** “Since you started using PDMonitor^®^, do you consider that your condition has improved, worsened, or remained unchanged?”

**Choices**	**Answers (%)**
Significantly improved	11.1
Improved	33.3
Remained unchanged	37.0
Worsened	14.8
Significantly worsened	3.7
Didn't answer	0

**Table 5:** “To what degree do you agree that the use of PDMonitor^®^ has improved the communication with your physician?”

**Choices**	**Answers (%)**
Strongly agree	63.0
Agree	18.5
Undecided	3.7
Disagree	14.8
Strongly disagree	0
Didn't answer	0

## 4 Discussion

In this study, the control of motor symptoms in patients with PD was examined over a two-year observation period. The investigation centered on patients who underwent traditional medical examination combined with a comprehensive analysis of objective ambulatory measurements collected at non-clinical settings. The impact of telemonitoring for continuous home monitoring on the management of PD was evaluated.

Real-world evidence of this new treatment paradigm showed a significant improvement in the percentage of OFF time over the 24-month study period (Figure 1A). The observed decrease in OFF, accompanied by a trend of increasing ON time (Figure 1E), suggests a positive influence of telemonitoring on motor symptom control. Increasing the number of patients with improved or stable symptom manifestation, as reflected by the absence of statistical differences and significant trends in device-reported UPDRS and DYS scores (Figures 1G, J), further emphasizes the reliability of the telemonitoring approach in PD management. Further investigations are warranted to explore limited exacerbation in dyskinesia and UPDRS, as indicated by only a subset of participants (Figures 1I, L). In cases where patients experience worsening symptoms, the referral to advanced therapies, such as DBS, should be considered (16).

The clinical significance of these findings is enhanced when considering that the motor function is usually worsened significantly with time in patients with PD. Previous studies suggest that annual rates of progression of the total UPDRS score range from 7.8 to 14 points, and of the UPDRS III (motor) score from 5.2 to 8.9 points (17, 18). Moreover, the worsening is faster during the first years of disease and the increase over time is independent of sex and age (19, 20). The late stages of Parkinson's disease are marked by a progressive decline in both physical and cognitive function, leading to a substantial reduction in quality of life for individuals affected by the condition (21) and placing significant strain on caregivers and healthcare systems (22, 23). Minimizing “OFF” time and maximizing “ON” time leads to an improved quality of life for patients, with optimum cognitive and mental health (24). Improved motor function and reduced motor fluctuations are the key considerations to be taken into account in PD treatment to minimize potential side effects such as postural abnormalities and freezing episodes, which are associated with an increased risk of falls (25, 26). Telemonitoring emerges as a valuable tool for healthcare professionals, facilitating the optimization of medication management and enabling timely adjustments to the treatment plan (27). When symptoms are effectively controlled, the necessity for follow-up consultations can be diminished, thereby achieving treatment waning (13).

The frequency of therapy adjustments and the subsequent increase in dopaminergic burden underscore a critical consideration in treatment approaches. In this study, the average change in treatment was minimal, resulting in an LEDD of 255.6 mg (from 799.6 to 1,055.2 mg) (Table 1). Although there are periods lasting up to several years in which pharmacological treatment could extremely efficiently control symptoms, the majority of PD patients will experience ineffective symptom management during disease course (28). For instance, the patient illustrated at Figure 2 shows improved symptoms control, which, however, was accompanied with an increased L-Dopa of about 800 mg during the 2 year follow-up period. This highlights that there is an ongoing challenge in maintaining optimal symptom control without increasing dopaminergic burden. While effective symptom management is a desirable outcome in PD management, healthcare practitioners should balance the therapeutic benefits of dopaminergic medications with the potential risks of increased burden and side effects (29). This emphasizes the importance of continually reassessing treatment strategies through the use of telemonitoring to address evolving patient needs and disease dynamics.

The findings of this study also demonstrated that telemonitoring not only aids in objective symptom assessment but also contributes to a positive subjective experience for patients, potentially influencing adherence to treatment plans. It should be noted that the prevalence of significant medication non-compliance in PD is high and it is linked to reduced quality of life and heightened severity of both motor and non-motor complications (30, 31). Therefore, the reported improvement in the perceived medication effectiveness underscores the potential of telemonitoring in optimizing treatment strategies by improving treatment adherence and satisfaction of patients about their disease status (Table 2). The questionnaire-based assessment of patient satisfaction revealed a notable increase in the percentage of satisfied and very satisfied patients following telemonitoring (Tables 3, 4). Perceived advantages of remote monitoring, as reported by a significant majority of participants, also include improved communication with physicians (Table 5), highlighting an improved, patient-centered approach to care. In accordance with findings in the literature, considerable interest of employing telemonitoring is observed for patients as well as healthcare professionals, with noteworthy levels of satisfaction reported by both parties (32). The quality of care administered to PD patients through telemedicine is deemed comparable to that of in-person care, albeit a preference for a hybrid approach combining telemonitoring and in-person visits has been expressed by patients (33).

Despite the promising findings, this study has several limitations. The small sample size may limit the generalizability of the results, and further research with larger cohorts is warranted to validate the observed trends. Additionally, the exclusion of patients with advanced therapies such as DBS and infusion pumps may limit the applicability of the findings to this specific subgroup. Future studies, considering factors such as device adherence, should explore the long-term sustainability of telemonitoring benefits. This study relied on device-reported outcomes to assess symptoms, which does not allow a direct comparison with previous studies that used standard clinical scales (e.g., UPDRS). Future research comparing telemonitoring with traditional in-person care approaches and assessing the cost-effectiveness of telemonitoring interventions would provide additional insights into its broader implications. Additional studies are required to quantify the net effect of telemonitoring on symptoms control. Controlled longitudinal evaluation for longer observation period could assess whether a different symptoms trajectory exists between patients undergoing traditional practice and people undergoing telemonitoring.

In conclusion, implementing telemonitoring could lead to more efficient use of healthcare resources. By reducing the need for frequent in-person visits, clinicians can allocate their time more effectively, focusing on patients who require immediate attention while remotely monitoring others. The study indicates that this paradigm shift in PD improved patient satisfaction with their treatment and communication with healthcare providers. This enhanced engagement can lead to better adherence to treatment plans and more active participation in managing the disease, ultimately improving outcomes. Additionally telemonitoring facilitates better disease management and potentially reduces the frequency and severity of symptom exacerbations, which in turn can alleviate the burden on caregivers. Consequently, this may result in an improved quality of life for both patients and their families.

## Data availability statement

The data analyzed in this study is subject to the following licenses/restrictions: the datasets generated for this study are available on request to the corresponding author. Requests to access these datasets should be directed to g.rigas@pdneurotechnology.com.

## Ethics statement

Ethical review and approval was not required for the study on human participants in accordance with the local legislation and institutional requirements. Written informed consent from the patients/participants or patients/participants' legal guardian/next of kin was not required to participate in this study in accordance with the national legislation and the institutional requirements.

## Author contributions

SKoni: Writing – original draft, Writing – review & editing, Methodology, Conceptualization, Investigation, Supervision. AA: Conceptualization, Investigation, Methodology, Supervision, Writing – original draft, Writing – review & editing. PZ: Conceptualization, Investigation, Methodology, Supervision, Writing – original draft, Writing – review & editing. CS: Conceptualization, Investigation, Methodology, Supervision, Writing – original draft, Writing – review & editing. GT: Conceptualization, Investigation, Methodology, Supervision, Writing – original draft, Writing – review & editing. GX: Conceptualization, Investigation, Methodology, Supervision, Writing – original draft, Writing – review & editing. KT: Conceptualization, Investigation, Methodology, Supervision, Writing – original draft, Writing – review & editing. NK: Formal analysis, Software, Visualization, Investigation, Methodology, Writing – original draft, Writing – review & editing, Data curation. FK: Data curation, Formal analysis, Investigation, Methodology, Software, Visualization, Writing – original draft, Writing – review & editing. AN: Data curation, Formal analysis, Investigation, Methodology, Software, Visualization, Writing – original draft, Writing – review & editing. SKont: Data curation, Formal analysis, Methodology, Software, Visualization, Writing – original draft, Writing – review & editing, Validation. GR: Data curation, Formal analysis, Investigation, Methodology, Project administration, Software, Supervision, Visualization, Writing – original draft, Writing – review & editing.
